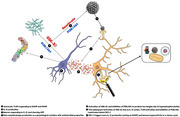# Neuroimmune Dysregulation in Early‐Onset Alzheimer's Disease: Astrocyte‐Mediated Innate Immune Hyperactivation Drives Pathogenic Feedforward Loops and Amyloid‐Beta/Tau Pathology

**DOI:** 10.1002/alz70855_105195

**Published:** 2025-12-24

**Authors:** Mohammad Walid, Haneen Beshr El‐Alfy, Rahma Mohammed Ibrahim, Mohammad Abdulhalim Ahmed, Roaa Salah Hassan

**Affiliations:** ^1^ Faculty of Medicine, Arish University, Arish, North Sinai, Egypt

## Abstract

**Background:**

Early‐onset Alzheimer's disease (EOAD) arises from genetic and environmental factors; however, therapies targeting presumed pathogenic processes remain ineffective. This gap highlights the need to reassess traditional pathogenesis models and investigate alternative mechanisms.

**Method:**

A comprehensive review of MEDLINE, PsycINFO, and Cochrane Central Register through January 2025 was conducted, supplemented by analyses of genetic/protein databases and molecular studies using human‐induced neural stem cells (hiNSCs).

**Result:**

Neuroimmune dysregulation emerges as central to Alzheimer's pathogenesis, surpassing amyloid‐beta (Aβ) and tau's roles. Traumatic brain injury (TBI) and neurotropic pathogens (HSV‐1, VZV) activate innate immunity via pattern recognition receptors (PRRs), inducing Aβ/tau pathology in hiNSC models. These stimuli engage pathogen‐associated molecular patterns (PAMPs) and damage‐associated molecular patterns (DAMPs), triggering self‐sustaining inflammation. Astrocyte‐derived interleukin‐1β (IL‐1β) stimulates neuronal Aβ production and activates glycogen synthase kinase‐3β (GSK‐3β) via Toll‐ like receptor (TLR) signaling, promoting tau hyperphosphorylation and impairing PI3K/Akt survival pathways. Critically, GSK‐3β facilitates HSV‐1 reactivation in models with latent HSV‐1 exacerbated by TBI, perpetuating TLR/IL‐1β feedforward cycles and increasing the amyloid burden in secondary analysis of hiNSCs studies. Aβ also functions as an antimicrobial peptide which reinforces the physiological role of Aβ but overproduced during immune hyperactivation which leads to the pathology of AD. Additionally, the protective gene LRRTM4, which regulates innate immunity via TLR4 and NF‐ kB pathways, has been implicated in modulating this immune response, suggesting that immune dysregulation may play a central role in AD pathogenesis. EOAD‐associated mutations (PSEN1, APP, APOE4) amplify astrocytic immune responses, triggering neuronal cytokine release and microglial activation, positioning Aβ as a downstream effector. Conversely, late‐onset AD (LOAD) stems from aging‐related immunosenescence, impairing microglial phagocytosis and Aβ/tau clearance. Hippocampal vulnerability in LOAD reflects metabolic‐oxidative stress interactions, while EOAD exhibits diffuse pathology from neuroinflammatory priming.

**Conclusion:**

Alzheimer's disease (AD) heterogeneity reflects distinct etiologies: EOAD is characterized by a hyperimmune state driven by astrocytic immune hyperactivity due to genetic susceptibility and environmental triggers, while LOAD arises from an aging‐related immunosenescence process. Both pathways ultimately converge on Aβ/tau deposition as secondary outcomes. This paradigm shift highlights the central role of innate immunity in AD pathogenesis, providing novel therapeutic targets to disrupt pathological cycles via immune modulation.